# Surgery improves survival rate in patients with Takayasu arteritis: a retrospective observational study

**DOI:** 10.3389/fcvm.2026.1782589

**Published:** 2026-07-29

**Authors:** Yining Fu, Tao Xiang, Yisen Yang, Jingya Zhou, Xiaoxiao Guo, Li Xu, Yuehong Zheng, Yuexin Chen

**Affiliations:** 1 Department of Vascular Surgery, Peking Union Medical College Hospital, Beijing, China; 2 Department of Epidemiology and Biostatistics, Institute of Basic Medical Sciences Chinese Academy of Medical Sciences, School of Basic Medicine Peking Union Medical College, Beijing, China; 3 Research Center, National Key Laboratory of Complex Severe and Rare Diseases, Peking Union Medical College Hospital, Beijing, China; 4 Department of Cardiology, Peking Union Medical College Hospital, Beijing, China; 5 Department of Anesthesiology, Peking Union Medical College Hospital, Beijing, China

**Keywords:** abdominal aorta, descending thoracic aorta, long-term outcomes, surgery, takayasu arteritis

## Abstract

**Objective:**

The long-term prognosis and optimal management for Takayasu arteritis (TA) patients with severe stenosis of the descending thoracic and abdominal aorta remain undefined. We compared the clinical outcomes of surgical intervention and medical management.

**Methods:**

Between January 2011 and January 2022, 78 TA patients with severe descending thoracic and abdominal aortic stenosis were enrolled at Peking Union Medical College Hospital. The cohort included the surgical group (*n* = 62) and the conservative treatment group (*n* = 16). Baseline characteristics were assessed to reduce selection bias. The primary outcome was all-cause mortality. Secondary outcomes were defined as the event-free survival and quality of life. Long-term follow-up of the clinical outcomes was also performed.

**Results:**

Surgical intervention significantly reduced all-cause mortality compared to conservative management (*P* < 0.001). Patients who underwent surgeries also exhibited improved quality of life regardless of their physical condition (*P* < 0.01) or psychological condition (*P* = 0.006). Postoperative reductions in systolic blood pressure (*P* = 0.033) and antihypertensive medication requirements (*P* = 0.015) were observed compared to medical management. In the surgical group, different surgical approaches presented with other complications, with the open surgical group having higher comorbidities within 30 days and a lower rate of long-term restenosis (*P* = 0.038).

**Conclusion:**

Surgical intervention significantly improves long-term outcomes in TA patients with severe stenosis of the descending thoracic and abdominal aorta.

## Introduction

TA is a rare chronic vasculitis that mainly involves the aorta and its main branches (1). The distribution of arterial lesions exhibits geographical and ethnic variations, with the descending thoracic and abdominal aorta representing the most frequently involved sites in Asian populations, followed by the aortic arch and its branches (2–4). Cardiovascular events are the leading cause of death (5, 6). Without intervention, outcomes are often unfavorable because of persistent hemodynamic compromise (7). To our knowledge, some reports have proven the efficacy of the surgical treatment, which may provide a more durable outcome. However, no large-scale retrospective cohort study has focused on the long-term consequences of medical vs. surgical approaches, and no criteria for indication for intervention have been established (8–10). Therefore, this study aimed to evaluate the clinical outcomes of different treatment strategies in TA patients with severe stenosis of the descending thoracic and abdominal aorta.

## Methods and analysis

### Materials and methods

**Figure 1:**
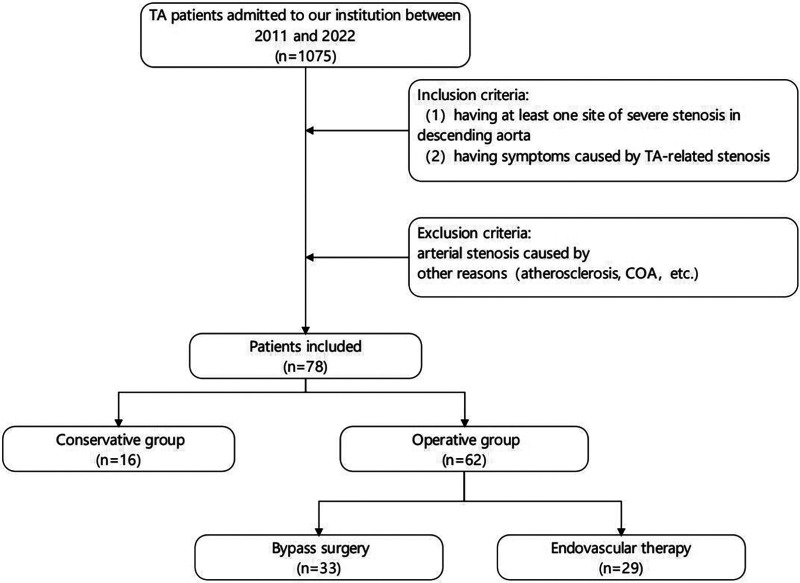
The flowchart of the patients included.

In this retrospective study, we collected data from inpatients at Peking Union Medical College Hospital from January 2011 to January 2022 with an expected follow-up of more than 3 years (shown in Figure 1). The inclusion criteria were as follows: (1) fulfilling the 1990 American College of Rheumatology criteria for the diagnosis of TA (11); (2) having at least one site with severe narrowing in the descending thoracic and abdominal aorta; (3) having symptoms caused by TA-related stenosis, such as uncontrolled hypertension, heart failure, renal ischemia, dizziness, visual disturbances, and claudication. Patients with arterial stenosis due to other reasons (such as typical atherosclerosis, congenital coarctation of the aorta, and anatomical abnormality) were excluded. Regarding the grouping methodology, patients who undergo open surgery at any point during their treatment are classified as open surgery cases, regardless of the number of subsequent intervention operations. Patients' informed consent was obtained. Postoperative patients were regularly followed up in the Vascular Surgery and Immunology Department to adjust medications. During follow-up, their blood pressure was measured at a local hospital and recorded using a questionnaire. The study has been registered on ClinicalTrials.gov (NCT07149805).

### Invasive interventions and medication

The primary indication for invasive interventions is arterial stenosis, as significant aortic stenosis can induce substantial hemodynamic compromise, leading to refractory hypertension and severe limb or end-organ dysfunction (e.g., claudication and renal insufficiency) (12). Our center employs diverse surgical approaches, including bypass surgery and endovascular therapy. The location and extent of the lesions determine the procedure. Angioplasty is suitable for focal stenoses, whereas diffuse long-segment coarctation or failed endovascular therapy necessitates extra-anatomic bypass (13). Although all enrolled patients met the surgical criteria, some opted for conservative management due to perceived procedural risks or economic constraints, consistent with patient-centered care models. Conservative therapy comprising glucocorticoids and conventional immunosuppressants to control inflammation, with biological agents added for refractory or relapsing disease, serves as a foundational treatment and may mitigate disease progression when invasive interventions is declined (14–17).

Treatment decisions were individualized based on patient-specific characteristics. Surgical principles dictate that bypass grafts should be anastomosed to disease-free proximal and distal vessels adjacent to stenotic/occlusive segments, with procedural selection tailored to each case. For thoracoabdominal aortic involvement, bypass grafts typically originate from the ascending aorta, a segment rarely affected by TA. Following median sternotomy and ascending aortic exposure, systemic heparinization was performed. A vascular graft (typically Dacron or ePTFE) was then anastomosed to the clamped ascending aorta, tunneled through the diaphragm and lesser omentum posterior to the stomach, and terminated at the uninvolved distal aorta. Extensive lesions required combined procedures (18, 19). For carotid arterial reconstruction, we used a prosthesis from the ascending aorta to the carotid artery (diameter 6/7 mm) or a prosthesis graft. In patients with affected renal arteries, the saphenous veins were used to bypass the infrarenal aorta, which may be associated with a lower probability of restenosis and anastomotic disruption.

### Endpoints and follow-up

The primary endpoint was the all-cause mortality rate. The secondary endpoints were event-free survival and quality of life assessed at the final follow-up. Health status was quantified using the Minnesota Living with Heart Failure Questionnaire (MLHF-Q) during hospitalization and follow-up periods (20). Therapy efficacy presented with symptom evolution, blood pressure control, and adjustments to antihypertensive medication regimens (dosage/type). Patients underwent follow-up from discharge until study termination or death. Those unable to attend our center completed assessments remotely via electronic questionnaires after a local hospital examination or structured telephone interview.

### Definitions

Significant stenosis was defined as >70% diameter reduction on Computed Tomography Angiography (CTA) or Digital Subtraction Angiography (DSA) or >20 mmHg pressure gradient across the stenosis by intraoperative angiography and manometry from the thoracic aorta to the end of the abdominal aorta (21).

Procedural success was defined as residual stenosis <50% of the treated lesion without flow-limiting dissection (13). Restenosis was defined as imaging-confirmed recurrent luminal narrowing of the treated or reconstructed vessel, irrespective of symptom status (22).

Complications were divided into early complications (within 30 days after invasive interventions) and late complications (>30 days after invasive interventions). Complications were defined as restenosis, bleeding, stroke, and/or other complications within 30 days of intervention and were not attributable to another condition.

Blood pressure measurement (Supplementary Figure S1) (23)

1. In patients without subclavian artery (SCA) involvement, a higher value in the arms was analyzed.
2. In patients with unilateral SCA involvement, the reading from the unaffected side was collected.
3. In patients with bilateral SCA stenosis, the central blood pressure was recorded to assess the true underlying blood pressure.

Blood pressure control was classified as (23)

1. Controlled: systolic blood pressure (SBP) <140 mmHg and diastolic blood pressure (DBP) <90 mmHg.
2. Improved: SBP ≥140 mmHg, but decreased by ≥20 mmHg and/or DBP ≥90 mmHg, but decreased by ≥10 mmHg.
3. Failed: did not meet the above criteria.

Disease activity (26)

1. Clinical symptoms: the presence of new or worsening systemic symptoms (fever, musculoskeletal complaints) or vascular ischemia (claudication, diminished or absent pulse, bruit, carotidynia, or asymmetric blood pressure).
2. Laboratory indicators: an elevated erythrocyte sedimentation rate (ESR) (≥20 mm/1st h) and/or C-reactive protein (CRP) (≥5 mg/L) in the absence of infection.

### Statistical analysis

Statistical analysis was performed using the SPSS software (SPSS 26.0, SPSS Inc.). If normality assumptions were satisfied for continuous data, means ± standard deviations (mean ± SD) were reported, and a *t*-test or ANOVA was used. For non-normal data, which were presented as the median (P25, P75), the Mann–Whitney *U*-test was employed. Categorical data were expressed as proportions and examined using the chi-square test or Fisher's exact test. For all-cause death and the combined endpoints, survival curves were calculated using Kaplan–Meier estimates. *P* < 0.05 was considered statistically significant.

## Result

### Baseline features of the patients

Seventy-eight eligible patients were included in this analysis. Tables 1, 2 present the baseline characteristics of the cohort, which included 62 patients in the surgical group and 16 in the medical therapy group. Within the surgical group, the patients were stratified into two subgroups based on the intervention modality: open surgery (*n* = 33) and endovascular repair (*n* = 29). No significant intergroup differences were observed in the baseline characteristics between the surgical approaches.

**Table 1:** Baseline characteristics of patients with severe stenosis in descending thoracic and abdominal aorta (*n* = 78).

Variable	Total (*n* = 78)	Operative (*n* = 62)	Conservative (*n* = 16)	*P*
Demographics				
Sex (Female)	66 (81.3)	53 (85.5)	13 (81.3)	.68
Height, cm	160(±8.6)	160(±7.8)	158(±11)	.27
Weight, kg	57(±12.4)	57(±12.5)	55(±12)	.51
Age^a^, years	32(±13)	31(±13)	33(±16)	.68
Age of onset^a^, years	25(±11)	24(±11)	24(±11)	.86
Diagnostic delay^a^, years	1 (0.5,10)	1 (0.5,5.2)	8.4 (1,14.3)	.26
Length of stay^a^, days	21 (12, 28)	23 (14,28)	17 (10,26)	.09
Cardiovascular risk factors				
Smoke	5 (7.8)	4 (6.3)	1 (6.3)	.97
Drink	2 (3.1)	2 (3.2)	0 (0)	.47
Hypertension	69 (90.6)	55 (88.7)	12 (75.0)	.89
Diabetes	3 (4.7)	3 (4.8)	0 (0)	.37
Comorbidity				
Heart-related	17 (21.8)	12 (19.4)	5 (31.3)	.31
Head-related	9 (11.5)	8 (12.9)	1 (6.3)	.46
Renal dysfunction	5 (6.4)	2 (3.2)	3 (18.8)	.15
Hepatitis	3 (3.1)	2 (3.2)	1 (6.3)	.58
Tuberculosis	10(12.5)	8(12.9)	2(12.5)	.96

a Data are presented as mean ± SD, median (IQR), or *n* (%).

**Table 2:** Lesions location and classification of patients.

Variable	Total (*n* = 78)	Operative (*n* = 62)	Conservative (*n* = 16)	*P*
Lesion site				
Descending thoracic aorta	16 (20.5)	11 (17.7)	5 (31.3)	.10^†^
Suprarenal aorta and renal artery	42 (53.8)	33 (53.2)	9 (56.3)	
infrarenal aorta	16 (20.5)	14 (22.6)	2 (12.5)	
Thoracic and Abdominal aorta	4 (5.1)	4 (6.5)	0 (0.0)	
Arterial territory				
Pulmonary artery	3 (3.8)	2 (3.2)	1 (6.3)	.42
Coronary artery	8 (10.2)	5 (8.1)	3 (18.8)	.26
Ascending aorta	12 (15.4)	8 (12.9)	4 (25.0)	.32
Aortic arch	17 (21.8)	12 (19.3)	5 (31.3)	.28
Thoracic aorta	45 (57.7)	38 (61.3)	7 (43.8)	.19
Abdominal aorta	59 (88.4)	54 (87.1)	15 (93.8)	.50
Renal artery	45 (57.7)	34 (54.8)	11 (68.8)	.57
Lower limb artery	9 (11.5)	7 (11.3)	2 (12.5)	.82
Upper limb artery	2(2.7)	2(3.2)	0(0.0)	.42

The Numano classification was developed to evaluate the pattern of vascular involvement.

Vc, V with coronary artery involved; Vp, V with pulmonary artery involved.

† Represents Fisher's exact test.

Table 3 presents the baseline laboratory findings. The conservative group demonstrated significantly higher levels of acute-phase reactants than the surgical group, with a significantly elevated erythrocyte sedimentation rate (ESR) (*P* = 0.038). Although the conservative group showed numerically higher values in cardiac and renal function markers (creatinine, myocardial enzymes, and N-terminal pro-B-type natriuretic peptide), no significant intergroup differences were observed at baseline. Medication profiles were clinically comparable between the groups, except for statin use (*P* = 0.002) (Table 4).

**Table 3:** The baseline laboratory findings.

Variables	Operative	Conservative	*P*
Creatinine (*μ*moI/L)	83.43	127.71	.27
cTnl(μg/L)	0.17	0.46	.47
NT-proBNP(ng/L)	2,118.60	3,896.67	.55
ESR (mm/1st hour)	10.93	29.80	.038*
hsCRP	5.01	15.31	.089

ESR, erythrocyte sedimentation rate; hsCRP, high-sensitivity C-reactive protein.

cTnl (μg/L): Cardiac Troponin I.

* Represents statistical significance.

**Table 4:** Medication treatments in the baseline.

Treatment, *n* (%)	Operative (*n* = 62)	Conservative (*n* = 16)	*P*
Antihypertensive drugs	53 (85.5)	6 (37.5)	.47
No. of antihypertensive drugs			
<3	36 (58.1)	3 (18.8)	
≥3	17 (27.4)	3 (18.8)	
Corticosteroids	44 (71.0)	10 (37.5)	.64
Immunosuppressants	39 (62.9)	11 (68.8)	.47
Aspirin	21 (33.9)	3 (18.8)	.24
Anticoagulants	8 (12.9)	1 (6.3)	.28
Statins	10 (16.1)	3 (18.8)	0.002*

* Represents statistical significance.

### The details of the bypass surgery

Given the similar hemodynamic alterations observed in this cohort, we summarized the lesion sites and corresponding anastomosis sites for bypass surgery in Supplementary Table S1.

## Results

### Mortality

**Figure 2:**
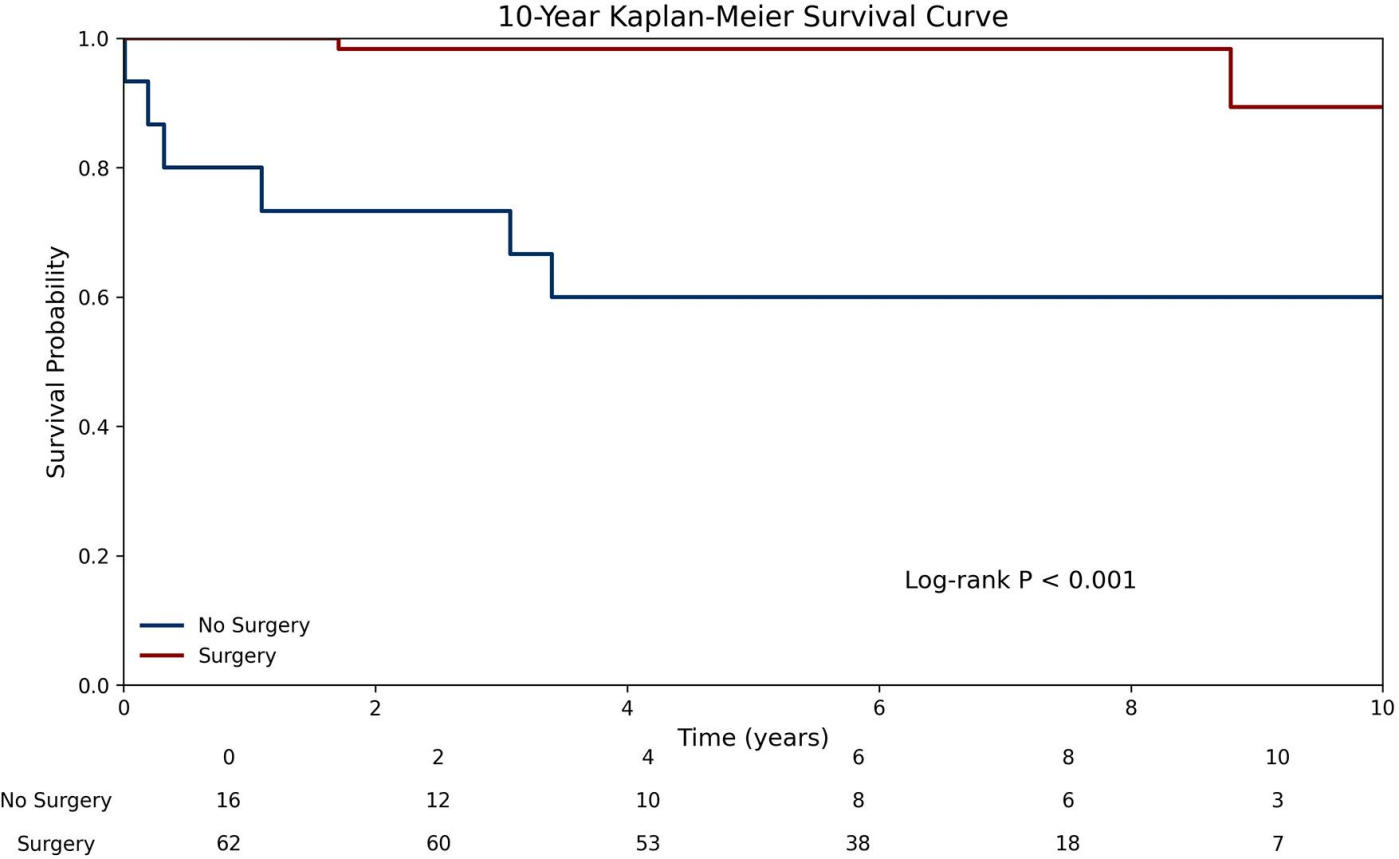
Kaplan–Meier survival curve for the study cohort in follow-up.

Of the 78 patients included, nine (11.5%) died during follow-up, and two (2.6%) were lost to follow-up. The major causes of death were renal failure and heart failure (Supplementary Tables S2 and S3). Compared with the surgical group, conservative treatment was associated with a significantly increased risk of all-cause mortality in follow-ups (*P* < .001) (shown in Figure 2).

Among patients who underwent invasive interventions, 33 received bypass surgery, 18 received stent implantation, and 11 received balloon angioplasty. During follow-up, death was uncommon in this subgroup, with only three deaths observed in total. One death occurred in each intervention modality group. The causes of death are summarized in Supplementary Table S2, and mortality according to intervention modality is shown in Supplementary Table S3. Kaplan–Meier analysis showed no statistically significant difference in overall survival among bypass surgery, stent implantation, and balloon angioplasty groups (log-rank *P* = 0.787; Supplementary Figure S3).

### Minnesota living with heart failure questionnaire (MLHFQ score)

Both groups demonstrated a reduction in the total MLHFQ score (indicating improved quality of life), with the invasive interventions group exhibiting a significantly greater improvement (*P* < .001). Physical condition scores improved in both groups during follow-up, although significantly more markedly in the invasive interventions group (*P* < .001). Regarding psychological conditions, the burden of psychological distress decreased in the surgical group but increased in the conservative group (*P* = .006) (Supplementary Table S4).

### Echocardiography

Echocardiographic follow-up data were available for 18 patients in the surgical group (Supplementary Table S5). Comparison with baseline measurements revealed favorable cardiac remodeling: although all values remained within normal limits, LVIDd and LV wall thickness decreased, while LVEF significantly increased post-treatment.

### Inflammatory indicators

The higher ESR in the conservative treatment group prompted us to examine the potential impact of elevated inflammatory marker levels on patient outcomes. Using regression models adjusted for age and sex (Supplementary Table S6), we assessed the relationship between surgical intervention/inflammatory markers and the outcomes. Invasive interventions was associated with a higher survival rate (*β* = 5.94, 95% CI: 1.1–32.4), whereas inflammatory markers did not significantly affect the prognosis (*β* = 0.29, 95% CI: 0.45–1.8).

### Medication

We further analyzed the changes in medication use before and after treatment among the 65 patients with available follow-up data (Supplementary Table S7). A significant reduction in antihypertensive medication use was observed in the invasive interventions group (*P* = .015). Specifically, 11 patients decreased the number or type of antihypertensive medications and 13 patients discontinued them entirely. Although the number of patients taking corticosteroids, immunosuppressants, and statins decreased, the number taking aspirin increased.

### Blood pressure control

In the invasive interventions group, the rates of hypertension control, improvement, and failure during the study period were 68.3%, 24.4%, and 7.3%, respectively. In contrast, none of the patients receiving conservative treatment achieved “control” status; 44.5% were classified as “improved,” while 55.6% failed to control their blood pressure. Overall, some degree of blood pressure improvement was observed in both groups. However, a significant improvement in systolic blood pressure was noted in the invasive interventions group (*P* = .033), whereas no significant difference in diastolic blood pressure was found between the two groups (Supplementary Table S8).

### Complications of interventions

The open surgery group had a higher rate of early complications (9 patients, 27.7%) than late complications (3 patients, 9%) during follow-up (Supplementary Table S9). Conversely, the interventional group experienced fewer early complications (1 patient, 3.4%) but more late complications (5 patients, 17.2%). Thus, short-term complications were more prevalent after open surgery, whereas long-term complications were more frequent after intervention. Critically, concerning long-term patency, restenosis (≥50% stenosis at the same site) occurred in only one patient (3%) in the open group and 11 patients (37.9%) in the interventional group. Ten patients with restenosis in the intervention group required repeat procedures. Freedom from reintervention was further evaluated according to intervention modality. Kaplan–Meier curves showed a significant difference in freedom from reintervention among patients undergoing bypass surgery, stent implantation, and balloon angioplasty (log-rank *P* < 0.001; Supplementary Figure S2).

## Discussion

This study evaluated the long-term prognosis of patients with TA presenting with severe stenosis in the descending thoracic and abdominal aorta and compared the outcomes across different treatment modalities. Previous research indicates that 18%–70% of patients with TA ultimately require surgical intervention (27, 28). However, owing to the rarity of TA, universally accepted surgical criteria are absent, especially for patients with critical luminal stenosis in the descending thoracic and abdominal aorta. The primary contribution of this study lies in demonstrating that the treatment choice significantly influences patient prognosis, thereby highlighting the critical need for evidence-based intervention strategies.

Surgical intervention, whether via endovascular therapy or bypass surgery, is generally considered beneficial for treating TA (9, 29). Our findings indicate that surgical management is associated with improved survival rates in patients with involvement of the descending thoracic and abdominal aorta (3). However, the different surgical approaches have distinct advantages and disadvantages (8). Endoluminal repair is often preferred for localized lesions because it is less invasive (27). Nevertheless, stenosis resulting from TA differs from atherosclerotic lesions; it requires higher pressure during balloon dilation, which increases the risk of vascular complications such as pseudoaneurysm formation, dissection, and arterial rupture (30). Moreover, TA-related concentric stenosis makes it difficult to achieve the desired therapeutic effect compared to eccentric stenosis typically seen in atherosclerosis (31). Conversely, the open surgical approach, which is potentially necessary for complex cases, is associated with higher technical demands, especially for multifocal disease, and carries a greater risk of procedural and perioperative complications, including postoperative anastomotic aneurysms and graft occlusion (31). Consistent with previous reports, our study observed that although patients undergoing open surgery experienced a higher incidence of early complications, they exhibited a lower rate of restenosis and a need for reintervention (6, 32, 33). Notably, the prevalence of arterial dissection and aneurysm in our cohort was lower than that reported in other studies. This discrepancy may reflect advancements in surgical techniques and/or a relatively short follow-up period.

Previous research has also indicated that surgical intervention can enhance cardiac function, which is similar to our results (34). The results of echocardiography were documented during the preoperative staging and follow-up for the 18 patients who underwent invasive interventions. Although echocardiographic measurements remained within normal ranges both before and after treatment, the surgical procedure likely contributed to the improved cardiac function. The deterioration of cardiac function may be due to increased peripheral resistance and myocardial fibrosis caused by renin-angiotensin-aldosterone system activation. Our study demonstrated that surgical intervention can ameliorate cardiac function, which is consistent with prior findings (35).

Hypertension is the most common feature observed in patients with TA. It is often related to renal artery stenosis and/or reduced aortic compliance from descending aortic issues (29, 36). Surgical intervention also demonstrated efficacy in ameliorating hypertension and reducing reliance on antihypertensive medications (29). Regarding medication usage, the post-surgical analysis revealed a decrease in the number of antihypertensive drug classes required compared with the preoperative levels.

Surgical intervention was associated with an improved quality of life in our patient cohort. Patients who underwent invasive interventions exhibited reduced physical and emotional symptom scores by 9.4 and 2.84 points, respectively. Notably, the conservative group showed a decline in physical function scores. We hypothesized that symptom improvement in non-surgical patients could stem from the formation of collateral vessels over the disease's long duration and possible adaptation to symptoms.

The timing of optimal intervention is crucial. Saadoun et al. (35) observed a sevenfold increase in complications when revascularization was performed during the inflammatory phase. In contrast, Fields et al. (25) found that although disease progression can occur postoperatively in patients with active disease, their long-term survival is not compromised. Our preferred approach was to schedule invasive interventions during periods of clinical stability (24). Nevertheless, invasive interventions may be necessary for patients in the active phase of their disease, particularly when medications fail to control symptoms or when disease activity represents a high risk of mortality or severe complications. Through careful assessment of the clinical symptoms and organ function, invasive interventions can be performed in certain urgent situations. We believe that rigorous perioperative care and pharmacological management can mitigate the adverse effects of disease activity on surgical outcomes.

The survival analyses according to intervention modality should be interpreted with caution. Although Kaplan–Meier curves were generated for bypass surgery, stent implantation, and balloon angioplasty, only three deaths occurred in the invasive intervention cohort, with one death in each subgroup. Therefore, the subgroup analysis was limited by the very small number of outcome events. Under such circumstances, the apparent ordering or terminal separation of Kaplan–Meier curves may be influenced by the timing of individual deaths, the number of patients remaining at risk, and the distribution of censoring during late follow-up. Thus, these curves should be regarded as descriptive summaries rather than definitive evidence of survival differences among intervention modalities (36).

Our study has several limitations. First, its single-center retrospective design may have limited the generalizability of our findings. Furthermore, due to the patient's specific clinical conditions, the allocation to endovascular or open surgery was not random, potentially introducing a selection bias. Although our study represents the largest cohort of TA patients with severe stenosis in the descending thoracic and abdominal aorta to date, the sample size remains relatively modest, which may have affected the statistical power. Consequently, prospective studies with larger cohorts are required to validate our results. Additionally, the study period spans over ten years, during which changes in medical practice and patient characteristics could have influenced the outcomes. Previous research suggests that postoperative complications should ideally be monitored for at least 20 years; therefore, our follow-up duration may have been insufficient to capture all the late events. Finally, the MLHFQ, while capturing relevant physical and emotional dimensions, has not been formally validated in TA patients, underscoring the need for disease-specific instruments in future studies.

However, the low incidence of TA makes it difficult to conduct large-scale randomized controlled trials. Nevertheless, as a major tertiary referral center in China for patients with TA who require revascularization, our institution has accumulated a substantial number of cases, which enabled us to assemble the largest cohort of TA patients with descending thoracic and abdominal aortic involvement reported to date.

## Conclusion

Our large-scale retrospective cohort study demonstrated that the surgical intervention improved the long-term outcomes in patients with severe descending aortic stenosis due to TA. These findings may offer clinical guidance for the development of standardized patient management strategies.

## Data Availability

The raw data supporting the conclusions of this article will be made available by the authors, without undue reservation.
