# Supplementary material for: Surgery improves survival rate in patients with Takayasu arteritis: a retrospective observational study
Source: Front Cardiovasc Med. 2026 Jul 29;13:1782589. doi: 10.3389/fcvm.2026.1782589 (PMC13461515; doi:10.3389/fcvm.2026.1782589)
Supplement: Supplementary file 1 [file Supplementaryfile1.docx]

Supplemental Table 1. Lesion site and surgical approach in patients with bypass surgery (n=33)

| Lesion Site | Number(n=33) | Surgical Approach | | | |
| --- | --- | --- | --- | --- | --- |
|  |  | A | B | C | D |
| Descending thoracic aorta | 4 | 4 | 0 | 0 | 0 |
| Suprarenal and interrenal aorta | 20 | 13 | 4 | 3 | 0 |
| Infrarenal aorta | 6 | 1 | 0 | 3 | 2 |
| Thoracoabdominal aorta | 3 | 2 | 1 | 0 | 0 |

A: Ascending aorta to abdominal aorta below the diaphragm

B: Thoracic aorta to abdominal aorta below the diaphragm

C: Suprarenal abdominal aorta to infrarenal abdominal aorta

D: Abdominal aorta to bilateral femoral arteries

Supplemental Table 2. Cause of death

|  | Total  (n=9) | Conservative  (n=6) | Operative  (n=3) | |
| --- | --- | --- | --- | --- |
|  |  |  | Endovascular therapy  (n=2) | Bypass surgery(n=1) |
| Heart failure | 3 | 2 | 0 | 0 |
| Renal failure | 4 | 2 | 2 | 0 |
| Heart failure and renal failure | 2 | 2 | 0 | 0 |
| Stroke | 1 | 0 | 0 | 1 |

Supplemental Table 3 Mortality by Surgery Type

| Surgery Type（n） | Death（n） | Death Rate (%) | Median Follow-up (years) |
| --- | --- | --- | --- |
| Bypass surgery (33) | 1 | 3 | 6.59 |
| Stent Implantation(18) | 1 | 5.6 | 7.31 |
| Balloon Angioplasty(11) | 1 | 9.1 | 5.04 |

Supplemental Table 4 Mean change in MLHFQ among patients (n=65)

| MLHFQ | Operative (n=56) | | | Conservative (n=9) | | | |  |
| --- | --- | --- | --- | --- | --- | --- | --- | --- |
|  | Baseline sample | Follow-up sample | Difference | Baseline sample | Follow-up sample | Difference | P value | 95% CI |
| Physical function | 27.1 | 17.2 | -9.9 | 22.7 | 21.3 | -1.4 | ＜0.01^*^ | 3-11 |
| Emotional score | 10.3 | 7.5 | -2.8 | 7.1 | 7.4 | 0.3 | .006^*^ | 0-5 |
| Total MLHFQ score | 37.4 | 24.7 | -12.8 | 29.8 | 28.6 | -1.2 | ＜0.01^*^ | 5-15 |

* Represents statistical significance.

95%CI: 95% confidence interval

Supplemental Table 5. Echocardiographic Changes in the Invasive Intervention Group (n=18)

|  | Preoperative | Postoperative | Difference | Normal value |
| --- | --- | --- | --- | --- |
| LVIDd | 48.8 | 44.7 | -4.1 | 35-55 |
| LVEF | 62.0 | 66.3 | 4.9 | 55-80% |
| LV wall thickness | 8.7 | 7.6 | -1.1 | 6-12 |
| E/A | 1.2 | 1.2 | 0.01 | ＞1 |

LVIDd: the left ventricular internal diameter in end diastole

LVEF: left ventricular ejection fraction

LV wall thickness: the left ventricular wall thickness

E/A: ratio of early diastolic mitral inflow velocity to atrial diastolic mitral inflow velocity

Supplemental Table 6. Associations of Invasive Intervention and Inflammatory Markers with Outcomes

| HR(95% CI) | Model 1* | Model 2* | Model 3* |
| --- | --- | --- | --- |
| Surgery | 5.94(1.1-32.4) | - | 9.0(1.1-69.4) |
| Inflammatory markers | - | 0.29(0.45-1.8) | 0.54(0.7-4.1) |

Model 1 describes the correlation between surgery and the outcomes

Model 2 describes the correlation between inflammatory indicators and the outcomes

Model 3 describes the correlation between surgery, inflammatory indicators, and the outcomes

*All models were adjusted for age and sex.

Notes: Adjusted model was applied using a linear regression.

Abbreviations: HR, hazard ratio; CI, confidence interval.

Supplemental Table 7. Changes in Medication Use

| Medication (n=65) | Operative(n=56) |  |  | Conservative (n=9) |  |  | P |
| --- | --- | --- | --- | --- | --- | --- | --- |
|  | In-hospital | Follow-up | Difference | In-hospital | Follow-up | Difference |  |
| Antihypertensive drugs | 37 | 27 | -10 | 6 | 9 | +3 | .015^*^ |
| No. of antihypertensive drugs |  |  |  |  |  |  |  |
| ＜3 | 21 | 22 | +1 | 3 | 5 | +2 |  |
| ≥3 | 16 | 5 | -11 | 3 | 4 | +1 |  |
| Corticosteroids | 35 | 20 | -15 | 7 | 5 | -2 | .97 |
| Immunosuppressants | 32 | 28 | -4 | 8 | 7 | -1 | .56 |
| Aspirin | 14 | 23 | +9 | 1 | 4 | +3 | .60 |
| Anticoagulants | 6 | 6 | 0 | 1 | 0 | -1 | .32 |
| Statins | 8 | 6 | -2 | 0 | 0 | 0 | .67 |

* Represents statistical significance.

Supplemental Table 8. Blood Pressure Changes at Baseline and Follow-up

|  | Operative (n=56) | |  | Conservative (n=9) | |  | p |
| --- | --- | --- | --- | --- | --- | --- | --- |
|  | Baseline | Follow-up | Difference | Baseline | Follow-up | Difference |  |
| Systolic blood pressure(mm Hg) | 156.6 | 126.1 | -32.7 | 156.1 | 138.5 | -7.2 | .033^*^ |
| Diastolic blood pressure(mm Hg) | 80.7 | 71.9 | -8.9 | 84.3 | 78.2 | -6.1 | .68 |

* Represents statistical significance.

Supplemental Table 9. Postoperative Complications in Invasive Intervention Group

|  | Total (n=62) | Bypass surgery (n=33) | Endovascular therapy  (n=29) |
| --- | --- | --- | --- |
| Complications within 30 days |  |  |  |
| Aneurysm, n | 1 (1.6%) | 0 | 1 (3.5%) |
| Pleural effusion, n | 2 (3.2%) | 2 (6%) | 0 |
| Delirium, n | 2 (3.2%) | 2 (6%) | 0 |
| Epilepsy, n | 1 (1.6%) | 1 (3%) | 0 |
| Myocardial infarction, n | 1 (1.6%) | 1 (3%) | 0 |
| Poor wound healing, n | 3 (4.8%) | 3 (9%) | 0 |
| Complications after 30 days |  |  |  |
| Hemorrhage, n | 1 (2.1%) | 1 (4%) | 0 |
| Pneumonia, n | 3 (6.3%) | 2 (8%) | 1 (4.3%) |
| Arterial dissection, n | 3 (4.2%) | 0 | 3 (8.7%) |
| Aneurysm, n | 1 (2.1%) | 0 | 1 (4.3%) |

^&^ All patients who required re-intervention had undergone endovascular therapy initially.

Supplemental Fig.1 Flowchart of Blood Pressure Measurement

Supplemental Fig.2 Kaplan-Meier Curve of Reintervention-Free Survival by Invasive Interventions Group

Supplemental Fig.3 Kaplan–Meier Curves of Overall Survival According to Invasive Intervention Strategy. Only three deaths occurred in the invasive intervention cohort, with one death in each group, these curves should be interpreted cautiously as descriptive subgroup analyses.
